# Electron Density Dual-energy CT for Liver Metastasis Detection Compared with Conventional and Virtual Monoenergetic Images at 40 keV

**DOI:** 10.2174/0115734056468428260624144643

**Published:** 2026-07-07

**Authors:** Cécile Chabrier, Maxime Leroy, Pr Olivier Ernst

**Affiliations:** 1 Digestive Imaging Unit, Center Hospitalier Universitaire de Lille, Lille, France; 2 SEED Unit (Statistics, Economic Evaluation, and Data Management), Centre Hospitalier Universitaire de Lille, Lille, France; 3 Digestive Imaging Unit, Center Hospitalier Universitaire de Lille, Lille, France

**Keywords:** Dual-energy computed tomography, Electron density, Liver metastases, Adenocarcinoma, Conventional images, Virtual monoenergetic reconstructions

## Abstract

**Introduction:**

To estimate the contrast-to-noise ratio (CNR) of liver metastases with electron density (ED) derived from dual-energy computed tomography and compare it with conventional and virtual monoenergetic images at 40 keV in both the unenhanced and portal-dominant phases.

**Methods:**

This observational retrospective, single-institution study included 32 patients with 49 liver metastases originating from digestive adenocarcinoma. All patients underwent dual-energy computed tomography (Spectral IQON^®^, Philips Healthcare) during unenhanced and portal-dominant phases. Conventional, virtual monoenergetic (VMI) images at 40 keV and ED images were reconstructed. The CNR of each metastasis was measured for each reconstruction in both the unenhanced and portal-dominant phases. Comparisons between techniques were conducted using linear mixed model regression.

**Results:**

Forty-nine liver metastases were included. For unenhanced acquisitions, ED demonstrated a statistically significantly higher CNR than both VMIs at 40 keV (mean difference 12.56 [95% IC, 10.17 to 14.95], *p* < 0.001) and conventional reconstructions (mean difference 12.83 [95% IC, 10.44 to 15.55], *p* < 0.001).

Similarly, in the portal venous phase, ED showed a significantly higher CNR compared with both 40 keV monoenergetic (mean difference 9.81 [95% IC, 7.42 to 12.20], *p* < 0.001) and conventional reconstructions (mean difference 16.62 [95% IC, 14.23 to 19.00], *p* < 0.001). Moreover, a significantly better CNR was observed between unenhanced ED and portal venous 40 keV VMI (14.4626 ± 7.5 *vs* 11.1810 ± 6.2, respectively; *p* = 0.007).

**Discussion:**

Few studies have investigated electron density. These findings suggest that the CNR of liver metastases with ED is better than conventional and VMIs at 40 keV reconstructions, in both the unenhanced and portal-dominant phases. The single-center design and the small sample size limit the generalizability of these results.

**Conclusion:**

Electron density provides a significantly better CNR for liver metastasis compared with both conventional and VMI at 40 keV reconstructions. Further studies are required to demonstrate that this gain in CNR improves the detection of hepatic metastases.

## 1 INTRODUCTION

19.3 million new cancer cases were estimated worldwide in 2020 [1] and survival among patients with liver metastases is significantly reduced [2]. Liver metastases are challenging to diagnose due to their often asymptomatic nature. Surgical resection is a potential curative option, but multiple locoregional treatments are also available for unresectable liver metastases arising from colorectal cancer [3]. Reliable and early screening methods are essential for patient survival.

Contrast-enhanced computed tomography (CECT) has been proven to be useful in detecting hypovascular hepatic metastases, particularly in the portal-dominant phase, which allows the detection of significantly more hypovascular hepatic metastases than any other phase does [4]. The beam-hardening effect is one of the physics-based artifacts of the CECT, originating from the polychromatic nature of the X-ray spectrum. It may mimic pathological findings or lead to misdiagnosis.

Dual-energy CT (DECT) technologies facilitate improved material differentiation and tissue characterisation by acquiring data at two distinct energy levels, thereby reducing physics-related artifacts [5, 6]. The recently introduced dual-layer detector computed tomography (DLCT) uses a spectral separator achieved at the detector level from a single X-ray source, consisting of two scintillator layers: the top layer absorbs lower-energy photons, whereas the bottom layer absorbs higher-energy photons [7].

Multiple post-processing techniques, such as virtual monoenergetic images and material composition images, such as electron density, are available because of their spectral properties. VMI allows reconstruction of images using a monoenergetic X-ray beam at a selected energy level. Previous studies have demonstrated improved detection of hypovascular metastases in low keV images [8, 9], particularly at 40 keV [10], because iodine attenuation increases as we approach the iodine K-edge value (33.2 keV). According to the Rose criterion, a lesion is detectable when its contrast-to-noise ratio exceeds approximately 5, meaning the signal must be at least five standard deviations above the background noise [11].

ED is an emerging imaging technique that displays the electron density of each voxel (*i.e*., the number of electrons within each voxel's volume) relative to that of water. This map is derived from a combination of Compton scattering and the photoelectric effect. A phantom study demonstrated a linear relationship between tissue ED, as measured by DECT, and density [12]. ED maps have been used for several decades in radiation therapy planning and have been further improved by the use of DLCT systems [13]. However, this technique is not yet widely adopted in diagnostic radiology. A limited number of studies have suggested a better visualisation of pulmonary embolism [14, 15], cervical disc herniation [16] or early-stage lung opacities in patients with COVID-19 pneumonia [17] using electron density.

We hypothesised that ED maps may improve the contrast-to-noise ratio of liver metastases, compared with conventional and monoenergetic at 40 keV images, in both the unenhanced and portal-dominant phases. The study aimed to compare these different imaging techniques for detecting hepatic metastases, both with and without iodinated contrast administration, in terms of CNR.

## 2 MATERIALS AND METHODS

This retrospective study was approved by our institutional review board, which waived the requirement for informed consent. This study was conducted in accordance with ethical standards and approved by the institutional review board.

### 2.1 Study Population

This was a retrospective observational, single-institution study. A total of 388 patients were identified in 2024 through the Radiology Information System at Lille University Hospital (France) using the keywords “CT and hepatic metastasis or secondary lesion”. The inclusion criteria were as follows: the presence of at least one liver metastasis larger than 15 mm; originating from a digestive adenocarcinoma; completion of both unenhanced and portal-venous phase CT examinations; and raw data available from a spectral CT scan (IQON^®^, Philips). The exclusion criteria were as follows: individuals under 18 years of age; a history of interventional radiology procedures; and cases where raw data were unavailable.

### 2.2 Dual-energy CT Protocol

All patients underwent both unenhanced and portal-dominant phase contrast-enhanced CT, using a dual-layer detector CT system (Spectral IQON^®^, Philips Healthcare). All scans were performed in helical mode with a pitch of 1.

CT scans were performed with patients in the supine position, covering the area from the top of the diaphragm to the symphysis pubis in a craniocaudal direction. Images were acquired using a tube voltage of 120 kVp, automated attenuation-based dose modulation (DoseRight, Philips Healthcare), a rotation time of 0.4 s, collimation of 64 x 0.625 mm, and a slice thickness of 1 mm. Raw data for each patient were stored in the Picture Archiving and Communication System (PACS).

The unenhanced phase was performed before intravenous administration of the iodine contrast agent. A dose of 1 mL/kg of Iomeron 400 mg/mL (Bracco) was administered via the median cubital vein at a rate of 3 mL/s using a power injector. Portal phase acquisition was performed using a bolus tracking technique, initiated 65 s after detection of an aortic peak enhancement of 180 HU.

### 2.3 CT Reconstructions

**Fig. (1):**
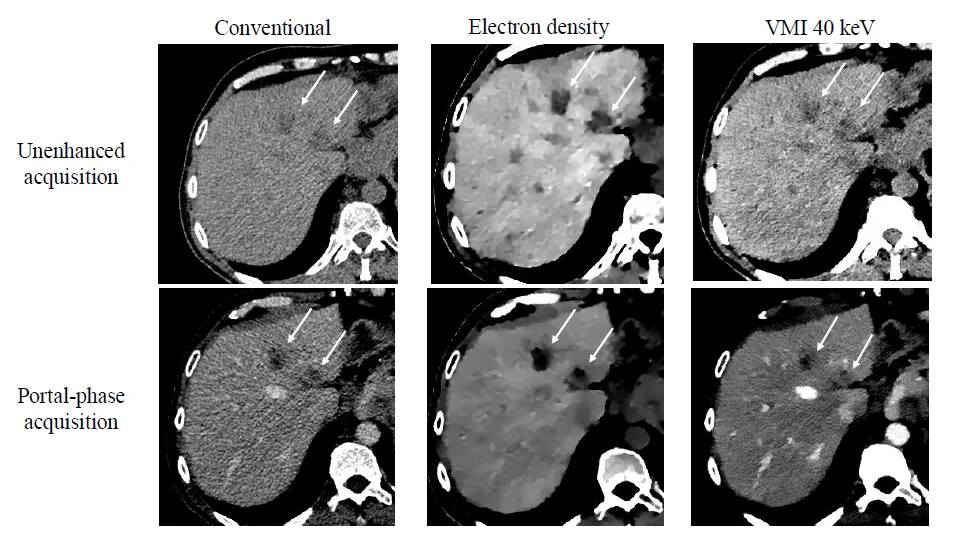
Liver metastasis (arrow) in a 57-year-old man with esophagogastric junction cancer.

**Fig. (2):**
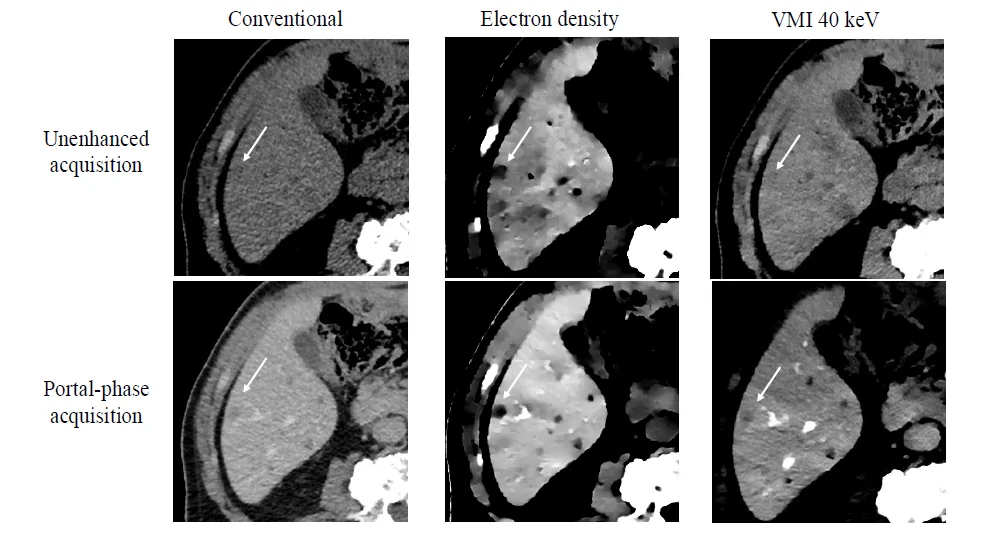
Liver metastasis (arrow) in a 81-year-old man with rectal cancer.

Conventional, ED, and 40 keV VMI reconstructions were generated from spectral data using a commercially available advanced visualization and analysis platform (IntelliSpace Portal 10.1; Philips Healthcare), employing the vendor’s standard reconstruction algorithms. Images were reconstructed with a slice thickness of 3 mm (Figs. **1**, **2**).

### 2.4 CNR Calculation Method

For each metastasis and the adjacent liver parenchyma at the same scan level, a homogeneous region of interest (ROI) was manually drawn in the shape of a cylinder with a minimum area of 0.50 cm^2^, avoiding blood vessels.

This ROI was automatically identical in size and location on conventional, 40 keV, and electron density images. Thus, the mean Hounsfield units (HUs) and mean percentage ED relative to water (%EDW) were automatically calculated for the same ROI.

The metastasis-to-liver CNR on conventional and virtual 40 keV monoenergetic images was calculated using the following formula [8]:


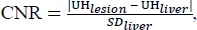


defined as the absolute difference between lesion and liver attenuation divided by image noise, measured as the standard deviation of liver attenuation.

For electron density reconstructions, CNR was calculated using the formula [8]:


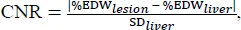


defined as the absolute difference between lesion and liver electron density values (expressed as a percentage relative to water), divided by image noise, measured as the standard deviation of liver attenuation.

### 2.5 CTDI

The volumetric CT dose index (CTDI_vol_) was extracted from the dose reports after each scan.

### 2.6 Statistical Analysis

Categorical variables were expressed as frequencies (percentages). Quantitative variables were expressed as the mean ± standard deviation (SD) or median [interquartile range (IQR)] in cases of non-normal distribution. Normality of data distributions was assessed both graphically and by using the Shapiro–Wilk test.

Comparisons between techniques were performed using a linear mixed model regression, including techniques of diagnosis of hepatic metastases as a fixed effect and metastasis as a random effect. The mathematical formulation of the model was: Y_jk_ = β_0_ + β_k_ × X_jk_ + u_j_ + ϵ_jk_, where j = 1, ..., 49 indexes metastases and k = 1, …, 5 indexes techniques (with the conventional unenhanced acquisitions technique as reference), and u_i_ ~ N (0, σ^2^_µ_) denotes the random intercepts for patients i, and ϵ_ij_ ~ N (0, σ^2^) is the residual error term. X_ijk_ is a set of dummy variables coding the technique, such that X_ijk_=1 if observation j corresponds to technique k, and X_ijk_=0 otherwise, with the reference technique implicitly defined when all indicator variables equal 0.

Post-hoc pairwise comparisons were conducted using linear contrasts. The normality and homoscedasticity of residuals were checked graphically.

All statistical tests were two-tailed, and a p-value < 0.05 was considered statistically significant. All analyses were performed using SAS software (version 9.4; SAS Institute Inc., Cary, NC, USA).

## 3 RESULTS

### 3.1 Patients

A total of 49 liver metastases from 32 patients (21 men and 11 women; range, 40-84 years; mean age, 61.6 ± 11.34 years) were included between January and December 2024. All patients underwent a dual-energy CT scan and had adenocarcinoma with liver metastases, which was diagnosed via surgery or biopsy, with the primary tumour located in the colon (n = 24), rectum (n = 3), gastroesophageal junction (n = 3), oesophagus (n = 1), or pancreas (n = 1).

### 3.2 CNR

Detailed measurements of the CNR are presented in Table **1**.

In unenhanced acquisitions, CNR did not differ significantly between 40 keV monoenergetic and conventional reconstructions (mean difference 0.28 [95% CI, -2.11 to 2.67], *p* = 0.81). However, electron density reconstructions demonstrated a significantly greater CNR than both 40 keV monoenergetic and conventional reconstructions (mean difference 12.56 [95%CI, 10.17 to 14.94], *p* < 0.001, and 12.83 [10.45 to 15.^22^], p<0.001, respectively).

In the portal venous phase, 40 keV monoenergetic reconstructions showed a statistically significantly better CNR than conventional reconstructions (mean difference 6.81 [95%CI, 4.42 to 9.^19^], < 0.001). While electron density reconstructions again demonstrated a significantly greater CNR than both conventional (mean difference 16.6 [95%CI, 14,23 to 19], < 0.001) and VMI at 40 keV reconstructions (mean difference 9,8 [95%CI, 7,42 to 12,^19^], < 0.001).

Additionally, unenhanced electron density reconstructions provided higher CNR than portal venous phase monoenergetic reconstructions at 40 keV (mean difference 3.28 [95%CI, 0.89 to 5.67], *p* = 0.0072) and conventional reconstructions (mean difference 10.08 [95%CI, 7.70 to 12.48], *p* < 0.001).

Finally, electron density reconstructions showed a significantly better CNR in the portal-dominant phase than in the unenhanced phase (mean difference 6.53 [95%CI, 4.14 to 8.92], *p* < 0.001).

### 3.3 CTDI

The mean volumetric CT dose index (CTDI_vol_) was 8.6 ± 3.3 mGy for unenhanced acquisition and 10.4 ± 3.6 mGy for portal-dominant phase acquisition (*p* < 0.001).

## 4 DISCUSSION

The liver is a frequent site for metastases, due to its dual blood supply (portal and arterial), with colorectal and pancreatic cancers being the most common primary sites responsible for hepatic metastases.

Multiple studies and reviews have demonstrated that DECT provides additional information beyond conventional CT, for example, iodine concentration and spectral curve slope analysis, which are valuable for tumor characterization [18]. DECT also provides post-processing maps, such as monoenergetic images or electron density.

Our study demonstrates a better CNR for liver metastases using ED reconstructions compared with conventional and virtual monoenergetic images at 40keV in both the unenhanced and portal-dominant phases. Applying the Rose criterion, which requires a minimum CNR of 5 for reliable lesion visualization, our findings demonstrate that unenhanced conventional and VMI at 40 keV reconstructions offer poor detectability, whereas electron density, both in unenhanced and portal phases, provides excellent CNR values, well above this threshold [19].

A particularly relevant finding for clinical practice is the better CNR of liver metastases in the unenhanced phase using ED reconstructions compared with 40 keV monoenergetic reconstructions in the portal venous phase. This has significant implications for patients unable to receive iodinated contrast agents, such as those with chronic kidney disease.

To our knowledge, the potential role of ED in diagnosing liver metastases has not been previously reported. Few studies have evaluated the role of ED. For instance, as Nagano *et al*. [20] reported a statistically significant lower ED in metastatic compared to non-metastatic lymph nodes in non-small cell lung cancer, and a quantitative analysis [21] revealed significant differences in ED between osseous metastases and normal bone. However, Qiu *et al*. found that the ED values did not significantly differ between metastatic and non-metastatic lymph nodes in patients with colorectal cancer [22].

The accuracy of the mean percentage electron density estimation relative to water derived from DLCT was previously established by Mei *et al*. [23].

All hepatic metastases in our study were considered hypovascular, consistent with their origin from primary neoplasms typically recognised as hypovascular [24].

It is important to acknowledge the non-linear, square root relationship between the signal-to-noise ratio and CTDI_vol_. In our study, CTDI_vol_ was significantly lower in the unenhanced phase compared with the portal venous phase. Although this dose difference may slightly underestimate the CNR in the unenhanced phase, the impact is considered minimal, estimated at approximately 10%.

ED reconstructions have several limitations. First, ED reconstruction algorithms differ depending on the vendor, leading to differences in their performance, precision, and level of noise introduced.

Secondly, despite a better CNR for hepatic metastases in ED reconstructions, the resulting images were visually “smoothed out”, characterised by indistinct organ contours, diminished contrast between structures, and some degree of blurring, which compromises the interpretation of the CT based only on this reconstruction.

**Fig. (3):**
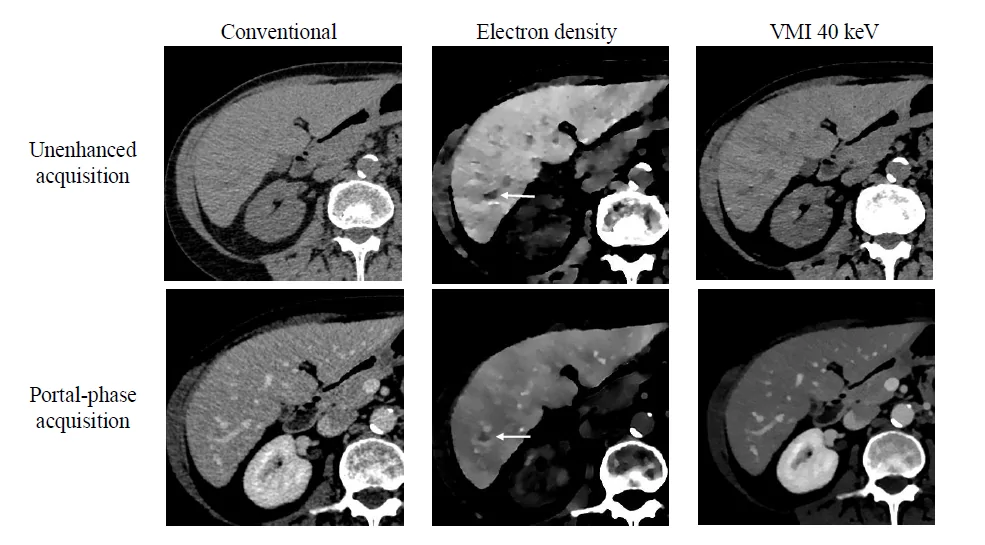
Example of a hypodense finding in a 62-year-old woman with sigmoid cancer that did not correspond to any visible abnormality on conventional or 40 keV VMI reconstructions.

Vessels were hypodense on non-contrast-enhanced imaging and demonstrated poor enhancement following contrast agent administration, complicating CT interpretation. We observed some hypodense structures on ED images, both in unenhanced and contrast-enhanced acquisitions, without corresponding findings in conventional or VMI images (Fig. **3**). This may be due to the noise reduction method used in the algorithm for calculating electron density. Indeed, applying noise reduction methods to lesions with a CNR below 5 can result in false-positive lesions [19].

**Fig. (4):**
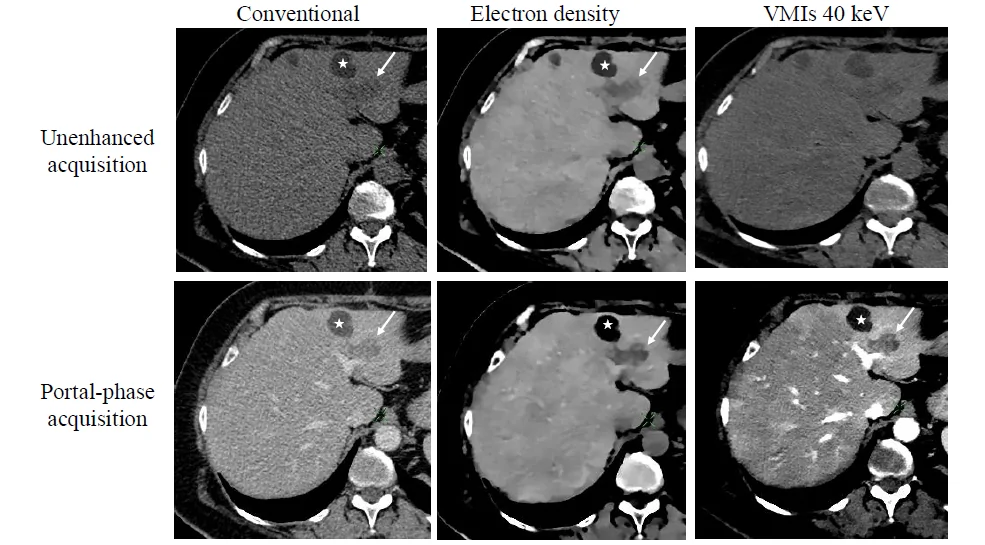
Liver metastasis (arrow) in a 57-year-old women with colonic cancer. A cystic lesion (star) is present next to the metastasis.

In the absence of iodine contrast enhancement, accurate differentiation of hypovascular metastases from other structures with similar ED, including cysts and vessels, remains challenging (Fig. **4**).

## 5 STUDY LIMITATIONS

This study had several limitations. First, its retrospective, single-centre design and the inclusion of a limited number of metastases (n = 49) may limit the generalizability of our findings. The sample size was small due to the preliminary nature of this study, which is the first to evaluate ED in detecting liver metastases.

The contrast-to-noise ratio results obtained with electron density are promising; however, they remain insufficient to demonstrate a true improvement in the detectability of liver metastases. Larger studies including more patients and multiple readers are required to confirm these findings.

Secondly, only metastases originating from adenocarcinoma have been evaluated. Third, we chose to include only lesions larger than 15 mm, as the partial volume effect is a well-known limitation that can impair the evaluation of lesions smaller than 15 mm [25]. Future studies should explore the potential of ED imaging in identifying sub-centimeter lesions to enable earlier treatment.

All scans in this study were performed on the Philips IQON spectral detector CT; results may not be directly reproducible on other platforms such as Siemens, Canon, or GE.

In this study, we compared the CNR of liver metastases using electron density reconstruction with conventional and monoenergetic reconstructions. It would be interesting to investigate the diagnostic performance of ED reconstructions compared with Magnetic Resonance Imaging (MRI), which is widely regarded as the reference standard for the detection of liver metastases [26].

While CNR provides a practical and widely used quantitative assessment of image quality, it does not fully capture the complexity of noise behavior [27], which may limit the interpretation of our results. Future work could incorporate more advanced noise modeling approaches to better characterize these underlying uncertainties.

## CONCLUSION

In conclusion, this study revealed a significantly better CNR for liver metastases using ED reconstructions compared with conventional and monoenergetic at 40 keV images. Our findings suggest that ED improves the visualization of liver metastases, provided that the image quality is optimized. Further studies are required to demonstrate that this gain in CNR improves the detection of hepatic metastases.

## Figures and Tables

**Table 1:** Comparaison of the contrast-to-noise ratio (CNR) of electron density (ED) with conventional and monoenergetic at 40 keV (VMI) reconstructions, in unenhanced and portal-dominance phases. Data are presented as mean ± standard deviation (SD).

**Variables**	**CNR ± SD**	-	-	-	-	-
**Unenhanced conventionnal**	1.6274 ± 0.9	**Unenhanced conventionnal**	-	-	-	-
**Unenhanced VMI 40 keV**	1.9067 ± 1.4	*p* = 0.81	**Unenhanced VMI 40 keV**	-	-	-
**Unenhanced ED**	14.4626 ± 7.5	*p* < 0.0001	*p* < 0.0001	**Unenhanced ED**	-	-
**Portal conventionnal**	4.3741 ± 2.2	*p* = 0.0243	*p* = 0.0428	*p* < 0.0001	**Portal conventionnal**	-
**Portal VMI 40 keV**	11.1810 ± 6.2	*p* < 0.0001	*p* < 0.0001	*p* = 0.0072	*p* < 0.0001	**Portal VMI 40 keV**
**Portal ED**	20.9932 ± 0.7	*p* < 0.0001	*p* < 0.0001	*p* < 0.0001	*p* < 0.0001	*p* < 0.0001

## Data Availability

The authors confirm that the data supporting the findings of this study are available within the article.

## LIST OF ABBREVIATIONS

CECT: Contrast-Enhanced Computed Tomography
DECT: Dual-Energy Computed Tomography
DLCT: Dual-Layer Detector Computed Tomography
keV: kilo-electronvolt
ED: Electron Density
VMI: Virtual Monoenergetic Images
CNR: Contrast-to-Noise Ratio
CTDI_vol_: Computed Tomography Dose Index
ROI: Region Of Interest
PACS: Picture Archiving and Communication System
%EDW: Electron Density Relative To Water
HUs: Hounsfield Units
SD: Standard Deviation
IQR: Interquartile Range
MRI: Magnetic Resonance Imaging

## STANDARDS OF REPORTING

STROBE guidelines were followed.
